# Effectiveness of an Educational Program on Awareness of Breast Cancer Risk Factors, Symptoms, and Barriers to Seeking Medical Help among Adolescent Omani School Students—An Interventional Study

**DOI:** 10.3390/curroncol30040314

**Published:** 2023-04-10

**Authors:** Khadija Al-Hosni, Moon Fai Chan, Mohammed Al-Azri

**Affiliations:** Department of Family Medicine and Public Health, College of Medicine and Health Sciences, Sultan Qaboos University, Muscat 123, Oman

**Keywords:** breast cancer, education, intervention, help-seeking behaviors, adolescents, Oman

## Abstract

Background and Aim: Women in Oman and low- and middle-income countries (LMICs) are usually diagnosed with BC at a younger age and more advanced stage, with poor five-year survival. This study aims to evaluate the effectiveness of breast cancer (BC) related educational programs among female Omani adolescents. Materials and Methods: Six female-only public schools were randomly selected from three governorates of Oman and assigned to the control or interventional group. An Arabic version of the Breast Cancer Awareness Measure questionnaire was used to evaluate students attending grades 10 and 11 at baseline (T0) and after 4 weeks (T1). After T0, the intervention group participated in a one-hour BC education program involving group discussions, a slideshow presentation, leaflets, and online access to program materials and videos. Non-parametric tests were used to compare scores between intervention and control groups and within each group across time (T0 vs. T1). Results: A total of 1106 students participated, of which 547 (49.5%) and 559 (50.5%) were allocated to the control and intervention groups, respectively. Recognition of BC risk factors (Z = 18.67; *p* < 0.001) and symptoms (Z = 20.01; *p* < 0.001) increased significantly in the intervention group between T0 and T1 and compared to the control group at T1 (U = 27.27; *p* < 0.001, and U = 25.75; *p* < 0.001, respectively). Anticipated time to seeking medical help (Z = 18.67; *p* < 0.001) and barriers to help-seeking (Z = 7.91; *p* < 0.001) decreased significantly between T0 and T1 in the intervention group and compared to the control group at T1 (U = 15.78; *p* < 0.001, and U = 3.44; *p =* 0.001, respectively). Conclusion: The program increased knowledge of BC risk factors and symptoms and promoted early medical help-seeking among Omani female adolescents. Healthcare strategic planners and policy-makers in Oman and low- and middle-income countries should consider incorporating cancer education programs in the national school curriculum to minimize delays in BC diagnosis and improve the survival rate.

## 1. Introduction

Breast cancer (BC) accounts for 10% of all cancers diagnosed annually and approximately 15% of all cancer deaths among women [1]. Despite global efforts to improve the detection and diagnosis of BC, nearly one-third of all women have either regional or distant metastasis at the time of diagnosis, most of whom reside in low- and middle-income countries (LMICs) [2]. Moreover, almost half of all women diagnosed with BC in LMICs are under 50, with a median age of 49–52 years, compared to women in more economically developed countries for whom the median age is 63 years [3,4]. Crucially, the mortality rate from BC in LMICs remains high despite the relatively low incidence rate compared to more developed countries [3].

Delays in cancer diagnosis are defined by a lengthy time interval between the appearance of the patient’s first symptom and their diagnosis and the subsequent start of treatment [5]. Recognition of BC symptoms and early medical help-seeking behaviors of affected patients can improve survival and prognosis [6]. Women who seek early medical help (i.e., within three months of the appearance of symptoms) have a greater chance of survival and cure compared to those who seek late medical help (i.e., three months or later after the appearance of symptoms) [7]. Delays in BC diagnosis usually occur because patients do not correctly identify BC symptoms or do not promptly act upon them and seek timely medical help [8,9].

Previous studies have shown that children and adolescents have low levels of cancer-related knowledge about risk factors and symptoms and infrequently engage in preventive behaviors [10,11]. Cancer education programs that seek to raise awareness of cancer risk factors and symptoms and promote early medical help-seeking represent an important initiative in primary cancer prevention and early diagnosis [12]. In particular, schools represent an effective setting to raise cancer awareness among adolescents and conduct other health promotion activities [13].

High school students (i.e., those between 12 and 19 years of age) are often deemed a suitable target for cancer education interventions, several of which have been found to result in improved knowledge and attitudes toward cancer prevention and the development of modifiable health behaviors in later life as the risk of cancer increases [10,11]. A recent systematic review concluded that interventional education programs are important to increase cancer knowledge among adolescent school students; as such, decision-makers should support the incorporation of cancer education within the curricula as part of their long-term cancer prevention efforts [14]. Different methods and materials have been used and have been shown to be feasible and acceptable in other studies to deliver cancer education to students, including face-to-face lectures, discussions, and the distribution of printed materials and videos [14]. However, it is unclear which specific method is most effective in increasing cancer knowledge and changing health behaviors [14].

Oman is a developing country in the Arabian Gulf region with a total population of 4.5 million; in terms of demographic structure, the population is youthful, with 35.7% being under 15 years of age [15]. In Oman, BC is the most commonly diagnosed cancer, accounting for 12.8% of all cancers and 21.2% of cancers affecting Omani women; moreover, its incidence almost doubled from 13.6 patients per 100,000 women in 1996 to 26.9 in 2015 [16]. Women with BC in Oman are usually diagnosed at a relatively young age (median age: 49 years) and more advanced stage at the time of diagnosis (i.e., stages III or IV), with a low five-year survival rate (63%) [17].

Previous studies have indicated that young Omani women have inadequate knowledge of BC symptoms [18,19]; moreover, such women do not prioritize seeking medical help and report several physical and emotional barriers to help-seeking despite awareness of the importance of an early BC diagnosis [18,20,21]. As such, researchers have concluded that there is an urgent need for cancer education programs to be included in local school curricula targeting female adolescents to improve their knowledge of BC symptoms and address help-seeking barriers to minimize delays in BC diagnosis. Accordingly, this study aimed to evaluate the effectiveness of an interventional BC education program to increase knowledge of BC risk factors and symptoms and reduce barriers to early medical help-seeking behaviors among Omani female adolescents.

## 2. Materials and Methods

### 2.1. Study Design and Sites

Oman is divided geographically into 11 governorates, with these governorates subsequently divided into 63 provinces. This study was conducted in three governorates of Oman, including Muscat, Al-Batinah, and Ad Dhakhiliyah. The selection of these governorates was based on convenience and was also intended to cover a variety of students from different geographic areas (i.e., urban, semi-urban, and rural areas). Two female public schools were selected randomly from each governorate and assigned to either the control or intervention group, with six schools being selected in total. Different schools were selected for the control and intervention groups to avoid undue peer influence on the student’s responses. 

Private schools enrolling non-Omani students and schools for students with special needs were excluded to avoid potential confounders and because most public schools in Oman follow a predetermined national curriculum set by the Ministry of Education (MOE). Adolescent Omani female students aged 15–17 years old and registered in grades 10 and 11 of the selected public schools were targeted for inclusion in the study. An invitation letter that included information regarding the purpose and design of the study was distributed to the students to pass on to their parents or guardians. Parents or guardians were asked to read the attached information form and to sign the consent form if they agreed for their children to participate in the study.

### 2.2. Sample Size Calculation

The power analysis for this study was based on a repeated measures design involving the pre/post comparison of two groups. Based on a previous study [10], the expected difference in BC knowledge levels between the intervention and control groups constituted a small effect size (0.10). Utilizing the Power Analysis PASS 2002 software version 2002 (NCSS Statistical Software LLC., East Kaysville, UT), the required sample for each group was found to be at least 500 in order to achieve 85% (between effect), 83% (within effect), and 85% (interaction effect) power at 5% alpha. Thus, considering an 8% drop-out rate per group, a total of 540 students were deemed necessary per group for a total sample size of 1080. 

### 2.3. Measurement Tool

First developed by the School of Cancer and Pharmaceutical Sciences, King’s College London, and the University College London in the UK, the Breast Cancer Awareness Measure (Breast-CAM) questionnaire is a validated, standardized tool to measure BC awareness in the general population [22,23]. The Breast-CAM was designed to be administered as a self-completed survey either online, by post, or under supervision during face-to-face or telephone interviews; however, completion of the questionnaire under supervision (i.e., either during face-to-face interviews or over the telephone) is recommended to yield the best quality data. In this study, we have administered the Breast-CAM under direct supervision. 

The Breast-CAM questionnaire is divided into four sections of multiple-choice questions. The first section assesses awareness of 11 BC symptoms or warning signs, including a lump or thickening in the breast tissue, a lump or thickening under the armpit, bleeding or discharge from the nipple, pulling or retraction of the nipple, changes in the position of the nipple, a rash on or around the nipple, redness of the breast skin, changes in the size of the breast or nipple, changes in the shape of the breast or nipple, pain in one of the breasts or armpits, and dimpling of the breast skin. Scores for this section are calculated by assigning one point to each correctly identified symptom, resulting in a total score ranging from 0 to 11.

The second section of the Breast-CAM includes nine close-ended questions assessing awareness of known BC risk factors, including a history of BC, using hormone replacement therapy (HRT), drinking alcohol, being overweight (body mass index of < 25 kg/m^2^), having a close relative with BC, having children later on in life or not at all, early menstruation, late menopause, and insufficient physical activity (<30 min of moderate physical activity five times a week). Scores for this section are calculated by assigning one point for each correctly identified risk factor, for a total score ranging from 0 to 9.

The third section of the Breast-CAM includes 10 items of preliminary breast health behavior questions to measure perceived barriers to seeking medical help for BC cancer symptoms or warning signs. These perceived barriers are further categorized into emotional (i.e., feeling embarrassed, scared, worried about what the doctor might find, or not feeling confident in talking about symptoms with the doctor), practical (i.e., being too worried about other things, too busy, or facing difficulty arranging transport to see the doctor), and service-related (i.e., facing difficulty making the appointment, being worried of wasting the doctor’s time, and finding it difficult to talk to the doctor) barriers. Scores for this section are calculated by assigning one point to responses of “yes” to each question. Finally, the fourth section of the Breast-CAM measures the anticipated time to consult a doctor for each of the 11 recognized BC warning signs (e.g., within two weeks, four weeks, six months, or later than six months). The questionnaire also included sociodemographic items. 

The test-retest reliability of the Breast-CAM over a two-week interval was found to be moderate to good for most items, with all correlations between 0.42 and 0.70. For the purposes of the current study, the Breast-CAM questionnaire was forward-translated into Arabic before being back-translated into English by researchers proficient in both languages. The internal reliability of the translated Arabic version of the Breast-CAM was high (Cronbach’s *α* = 0.817).

### 2.4. Data Collection

Data collection for this study occurred during the coronavirus disease 2019 pandemic. Students in both the intervention and control groups completed an online version of the Arabic Breast-CAM questionnaire under direct observation. Students were asked to complete the online Breast-CAM questionnaire at two different intervals: pre-intervention at baseline (T0) and four weeks post-intervention (T1). Three-digit codes were assigned to each student to guarantee anonymity.

After T0, students in the intervention group participated in a one-hour BC education program. The program included a slideshow presentation (PowerPoint, Microsoft Corp., Redmond, WA) and discussions focusing on BC incidence, pathophysiology, risk factors, warning symptoms, prevention strategies, screening, and the importance of early detection and seeking timely medical help upon first noticing BC symptoms. In addition, a leaflet was distributed with important information about cancer for the students to read. Finally, the students were advised to visit a webpage created by the research team to access the program materials and an informational video about cancer. Students in the control groups did not receive any cancer education or educational materials. Students in both groups were assessed at similar times for T0 and T1 and under direct observation.

### 2.5. Data Analysis

The students’ responses to the Breast-CAM questionnaire were scored at baseline pre-intervention (T0) and four weeks post-intervention (T1). The students’ responses were scored according to the instructions provided in the original Breast-CAM questionnaire [23]. Descriptive statistics (e.g., frequencies and percentages) were used to describe the demographic characteristics of each group. A chi-squared test was used to determine if there were any significant demographic differences between the groups to ensure homogeneity.

As the Kolmogorov–Smirnov test indicated that most of the outcomes were abnormal, further findings were analyzed using non-parametric tests. The Wilcoxon signed-rank test was used to compare scores between T0 and T1 for each group, while a Mann–Whitney U test was used to compare scores between groups at T0 and T1. For variables with binary outcomes (i.e., yes/no or agree/disagree responses), the McNemar test was used to examine intra-group differences at T0 and T1. In contrast, a chi-squared test was used to examine inter-group differences at T0 and T1. All statistical analyses were conducted using the Statistical Package for the Social Sciences (SPSS) software, version 26 (IBM Corp., Armonk, NY, USA), and the level of statistical significance was set at *p* < 0.05. No missing data were reported.

### 2.6. Ethical Considerations

This study was approved by the local medical research ethics committee of the College of Medicine & Health Sciences, Sultan Qaboos University, Muscat, Oman (MREC #2441). In addition, permission to conduct the study was obtained from the MOE, and each selected school’s principals were informed of the study in advance. Permission for individual students to participate in the study was obtained from each student’s parents and/or legal guardians.

## 3. Results

### 3.1. Sample Characteristics

A total of 1106 female students attending grades 10 and 11 of the selected schools agreed to participate in the study. There were 547 (49.5%) in the control group and 559 (50.5%) in the intervention group. Most students in the control group were aged 15 years (45.5%), while most in the intervention group were aged 16 (67.8%). A total of 92 students (16.8%) in the control group and 86 (15.4%) in the intervention group declared that they had health-related issues (e.g., obesity or diabetes), while 102 (18.6%) and 132 (23.6%) reported having relatives with cancer, respectively (Table 1). 

Only 20.8% and 22.2% of students in the control and intervention groups had heard of breast self-examination (BSE); moreover, 92.5% and 93.9%, respectively, had rarely or never undertaken BSE. Moreover, most students in the control and intervention groups did not feel confident that they would notice any changes in their breasts (49.5% and 47.8%, respectively), and few mentioned that they would visit a doctor if they were to notice such changes (3.7% and 2.1%, respectively). No significant differences between students in the intervention and control groups were observed regarding sociodemographic characteristics except for the age group (Table 1).

### 3.2. Recognition of BC Risk Factors

In the control group, mean total Breast-CAM scores for recognizing BC risk factors did not change significantly between T0 and T1 (2.16 ± 1.7 vs. 2.25 ± 1.6; Z = 1.87; *p* = 0.061). In contrast, mean total Breast-CAM scores in the intervention group increased from 2.13 ± 1.7 at T0 to 6.72 ± 1.4 at T1, indicating a significant improvement following participation in the BC education program (Z = 18.67; *p* < 0.001). Moreover, recognition of each specific BC risk factor increased significantly between T0 and T1 for the intervention group (Table 2). Furthermore, students in the intervention group demonstrated a significant improvement at T1 compared to the control group regarding overall recognition of cancer risk factors (U = 27.27; *p* < 0.001) and for each specific risk factor (Table 2).

### 3.3. Recognition of BC Symptoms 

As with recognition of BC risk factors, the control group demonstrated no significant change between T0 and T1 with regards to their mean total Breast-CAM scores for the recognition of BC symptoms (2.75 ± 2.55 vs. 2.83 ± 2.44; Z = 7.03; *p* = 0.081). However, mean total Breast-CAM scores in the intervention group increased significantly from 2.92 ± 2.7 at T0 to 8.15 ± 1.88 at T1 (Z = 20.01; *p* < 0.001). In addition, the intervention group showed a significant improvement between T0 and T1 for each specific BC symptom [Table 2]. A comparison of mean total Breast-CAM scores between groups at T1 indicated that the intervention group had significantly higher scores for recognizing BC symptoms than the control group (U = 25.75; *p* < 0.001). Moreover, students in the intervention group were significantly more able to correctly identify specific symptoms at T1 compared to the control group (Table 2).

### 3.4. Barriers to Seeking Medical Help

There was no significant change between T0 and T1 for the control group in terms of mean total Breast-CAM scores relating to perceived barriers to seeking medical help for BC symptoms (4.67 ± 2.6 vs. 4.65 ± 2.4; Z = 0.30; *p* = 0.765). In contrast, students in the intervention group significantly reduced mean scores for this section between T0 and T1 (4.59 ± 2.5 vs. 4.17 ± 2.2; Z = 7.91; *p* < 0.001). Moreover, they reported significant reductions between T0 and T1 with regards to the reporting of several specific barriers, including emotional barriers such as being scared (χ^2^ = 4.22; *p* < 0.001) or worried about what the doctor might find (χ^2^ = 3.08; *p* = 0.002); practical barriers such as having other things to worry about (χ^2^ = 3.20; *p* = 0.001), being too busy (χ^2^ = 6.87; *p* < 0.001), and having difficulty arranging transport (χ^2^ = 3.24; *p* = 0.001); and service-related barriers of being worried about wasting the doctor’s time (χ^2^ = 3.41; *p* = 0.001) and finding it difficult to talk to the doctor (χ^2^ = 4.25; *p* < 0.001) (Table 3).

Overall, the intervention group reported significantly lower mean total Breast-CAM scores at T1 than the control group (4.17 ± 2.2 vs. 4.65 ± 2.4; U = 3.44; *p =* 0.001). There were also significant differences between the two groups at the T1 stage with regards to the reporting of several specific barriers, including being scared (χ^2^ = 2.36; *p* = 0.018) or worried about what the doctor might find (χ^2^ = 2.03; *p* = 0.042), having other things to worry about (χ^2^ = 2.20; *p* = 0.028), being too busy (χ^2^ = 2.01; *p* = 0.044), facing difficulty arranging transport (χ^2^ = 2.17; *p* = 0.030), being worried about wasting the doctor’s time (χ^2^ = 2.55; *p* = 0.011), and finding it difficult to talk to the doctor (χ^2^ = 2.12; *p* = 0.034) (Table 3).

### 3.5. Anticipated Time to Seeking Medical Help

In terms of seeking medical help within the first two weeks of recognizing BC symptoms, no significant change was observed between T0 and T1 for the control group with regards to their mean total Breast-CAM scores (33.27 ± 16.8 vs. 33.49 ± 15.8; Z = 1.87; *p* = 0.061). Conversely, students in the intervention group demonstrated a significant increase in mean total Breast-CAM scores for this aspect between T0 and T1 (35.03 ± 15.8 vs. 48.29 ± 5.9; Z = 18.67; *p* < 0.001). Students in the intervention group also demonstrated a significant increase compared to the control group with regards to their mean total Breast-CAM scores at T1 (U = 15.78; *p* < 0.001). In addition, compared to the control group, students in the intervention group were significantly more likely to report at T1 that they would rapidly consult a doctor within two weeks upon recognizing all specific cancer symptoms (Table 3).

## 4. Discussion

The higher mortality rate and poor prognosis of BC in LMICs such as Oman are thought to be due to delays in diagnosis of more than three months between the recognition of symptoms and the time taken to consult doctors and access medical care [6,17,24]. Thus, improvements in knowledge of cancer risk factors and symptoms and help-seeking behavior modifications are required to overcome this problem via effective health promotion activities [21]. To our knowledge, this is the first interventional study conducted in Oman to evaluate the effectiveness of a cancer education program in enhancing awareness of BC risk factors and symptoms and reducing barriers to seeking medical help among female Omani adolescent students. In Oman, BC has been ranked as the most commonly diagnosed cancer, with affected women being diagnosed at a younger age and presenting at relatively advanced stages (i.e., stages III or IV) at the time of diagnosis [17,18]. Awareness of evidence-based cancer risk factors has been considered an important component of cancer control strategies [25]. 

In the present study, mean total Breast-CAM scores for recognizing BC risk factors improved significantly in the intervention group following participation in a BC education program. Knowledge regarding the impact of lifestyle changes (e.g., obesity, consumption of a high-fat diet, and smoking and alcohol consumption) in adolescence could impact young women and help them avoid BC risk factors and promote good health behaviors in adulthood [26]. The effectiveness of the current education program was supported by comparing the scores of the intervention group between T0 and T1 and by comparing the scores of the intervention and control groups at T1. Perceived risk of health problems, such as BC, has been regarded as a central construct in many models of health decision-making and, therefore, a useful target for behavior change interventions [14]. 

Previous research conducted in countries such as South Korea has revealed that education interventions can improve knowledge and attitudes toward cancer preventability up to three months post-intervention [27]. However, while education programs can result in short-term knowledge improvements in cancer prevention, their long-term effects on behavioral intentions and practices require further study [13]. Thus, such programs have been suggested to incorporate education booster sessions to assess and maintain long-term changes in cancer prevention behaviors [27]. In other countries such as New Zealand, researchers have recommended that the government provide adequate resources as part of a broader evidence-based public health program to increase cancer literacy and support preventive behavior changes in the general population [28].

Similar to BC risk factors, the intervention group in the present study reported significantly higher scores for the recognition of BC symptoms between T0 and T1 following participation in the educational intervention and compared to the control group at T1. These significant improvements were also observed for each specific BC symptom (e.g., a lump or thickening in the breast or under the armpit, bleeding or discharge from the nipple, pulling or retraction of the nipple, and changes in the position, size, or shape of the breast or nipple, etc.). Individuals who demonstrate greater knowledge of cancer symptoms are more likely to pay attention to such symptoms and demonstrate better intentions to seek timely medical help, particularly if they are female and highly educated [21,29].

Only 20.8% of students in the control group and 22.2% in the intervention group had heard of BSE, with the majority rarely or never undertaking this practice (92.5% and 93.9%, respectively). Moreover, most students in control (49.5%) and intervention (47.8%) groups reported that they did not feel confident that they would notice any changes to their breasts, while very few reported that they would visit a doctor were they to notice any such changes (3.7% and 2.1%, respectively). Previous studies conducted in Oman have reported several factors potentially contributing to delays in BC diagnosis, including misinterpretation of symptoms, being in denial, negative emotional perceptions related to BC symptoms, attitudes of fatalism, negative attitudes surrounding the idea of seeing a doctor, fears related to the consequences of a BC diagnosis and treatment, cultural beliefs and social stigma attached to the idea of being a cancer patient, and practical barriers to medical help-seeking, such as childcare responsibilities and lack of access to transport [8,18,21,30]. Indeed, early recognition of BC symptoms is futile without a corresponding intention to seek prompt medical help following symptom appraisal [18]. Addressing emotional or physical barriers to early medical-seeking behaviors and modifying negative beliefs or attitudes toward cancer can therefore help to promote early BC diagnosis and improve cancer survival [31]. The findings from our study showed that students in the intervention group reported significant reductions in various barriers to medical help-seeking behaviors, including reductions in specific emotional, practical, and physical barriers (e.g., being worried about what the doctor might find, being too busy, facing difficulty arranging transport, being worried about wasting the doctor’s time, etc.). Moreover, the education program was successful in promoting intentions to seek early medical help (i.e., consulting a doctor within two weeks) for each of the 11 BC-specific symptoms. However, there were no major changes in the barriers that were related to embarrassment as that might be difficult to be changed in a one-time short intervention [32].

### Limitations

This study has certain limitations. First, although the students were selected from 12 public schools in three separate governorates representing different geographic areas of Oman, a more nationally representative sampling of schools from all 11 governorates should be considered for future research. Second, we did not recruit students from private schools, although we believe this should not considerably impact the findings as the number of private schools in Oman is low compared to public schools. Third, the control condition was not an active or attention control. Finally, further research is recommended to determine the long-term effectiveness of school-based cancer education interventions and their impact on various lifelong cancer prevention behaviors. 

## 5. Conclusions

In conclusion, this study provides proof of the effectiveness of a school-based cancer education program in increasing knowledge and awareness of BC risk factors and symptoms and reducing emotional, physical, and practical barriers to seeking timely medical help for possible BC symptoms among female Omani adolescents. As the incidence and prevalence of BC are increasing in many LMICs, including Oman, with many patients being diagnosed at a young age and more advanced stage, incorporating similar health promotion initiatives within the public high school curriculum is becoming increasingly paramount. As such, healthcare strategic planners and policy-makers in Oman and other LMICs should consider training teachers to deliver such education to students. Future research should therefore consider seeking to identify the views of teachers and policy-makers in the MOE concerning possible obstacles to effective cancer educational programs in the national curriculum. Furthermore, future research is needed to determine the students’ effective health behavior in response to the employed type of interventional strategies, feedback on the program (intervention, evaluation, implementation process), and introduction of the BC screening program. Indeed, more future research is also needed to target emotional barriers by adding interventional content that normalizes feelings of embarrassment or teaches behavioral skills to improve self-efficacy for discussing breast health with providers.

## Figures and Tables

**Table 1:** Sociodemographic characteristics of the students (N = 1106).

Characteristic		Control (n = 547)	Intervention (n = 559)	χ^2^ (*p*-Value)
		n (%)	n (%)	
Age (years)	15	249 (45.5)	55 (9.8)	262.6 (<0.001)
	16	123 (22.5)	379 (67.8)	
	17	175 (32.0)	125 (22.4)	
Health issues	No	455 (83.2)	473 (84.6)	0.42 (0.516)
	Yes	92 (16.8)	86 (15.4)	
Replied Yes on health issues	Obesity	36 (39.1)	25 (29.1)	
	Respiratory	5 (5.4)	12 (14.0)	
	Blood disease	39 (42.4)	37 (43.0)	5.49 (0.240)
	Diabetes	7 (7.6)	7 (8.1)	
	Other	19 (20.7)	24 (27.9)	
Family member with cancer	No	334 (61.1)	313 (56.0)	4.44 (0.109)
	Do not know	111 (20.3)	114 (20.4)	
	Yes	102 (18.6)	132 (23.6)	
Degree of relative with cancer	First	20 (19.6)	37 (28.0)	7.81 (0.051)
	Second	11 (10.8)	10 (7.6)	
	Third	11 (10.8)	4 (3.0)	
	Other	58 (56.9)	80 (60.6)	
Have you ever undergone a breast examination?	No	540 (98.7)	532 (95.2)	1.10 (0.296)
	Yes	7 (1.3)	27 (4.8)	
Have you heard of breast self-examination?	No	433 (79.2)	435 (77.8)	0.30 (0.587)
	Yes	114 (20.8)	124 (22.2)	
How often do you check your breast?	Rarely or never	506 (92.5)	525 (93.9)	1.54 (0.673)
	Once every 6 months	17 (3.1)	14 (2.5)	
	Once a month	18 (3.3)	17 (3.0)	
	Once a week or more	6 (1.1)	3 (0.5)	
Are you confident you would notice a change in your breast?	Not at all	271 (49.5)	267 (47.8)	0.49 (0.926)
	Not very	142 (26.0)	146 (26.1)	
	Fairly	103 (18.8)	111 (19.9)	
	Very	31 (5.7)	35 (6.3)	
Have you seen a doctor about a change in your breast?	Yes	20 (3.7)	12 (2.1)	3.32 (0.190)
	No	80 (14.6)	71 (12.7)	
	Never noticed a change	447 (81.7)	476 (85.2)	

**Table 2:** Comparison of knowledge of breast cancer risk factors and warning symptoms between groups at baseline (T0) and four weeks later (T1) (N = 1106).

	Control (n = 547)			Intervention (n = 559)			Control vs. Intervention	
	T0	T1	T0 vs. T1	T0	T1	T0 vs. T1	T0	T1
Risk Factor ^^^	n (%)	n (%)	Test ^a^ (*p*-Value)	n (%)	n (%)	Test ^a^ (*p*-Value)	Test ^b^ (*p*-Value)	Test ^b^ (*p*-Value)
History of BC	135 (24.7)	137 (25.0)	0.30 (0.768)	148 (26.5)	459 (82.1)	17.52 (<0.001)	0.68 (0.494)	1.90 (<0.001)
Using HRT	98 (17.9)	101 (18.5)	0.56 (0.577)	112 (20.0)	391 (69.9)	16.70 (<0.001)	0.90 (0.369)	1.72 (<0.001)
Drinking alcohol	249 (45.5)	252 (46.1)	0.41 (0.680)	250 (44.7)	471 (84.3)	14.87 (<0.001)	0.27 (0.790)	13.43 (<0.001)
Being overweight	148 (27.1)	154 (28.2)	0.40 (0.688)	153 (27.4)	454 (81.2)	15.89 (<0.001)	0.12 (0.907)	17.73 (<0.001)
Family history of BC	186 (34.0)	194 (35.5)	1.60 (0.117)	207 (37.0)	456 (81.6)	15.47 (<0.001)	1.05 (0.293)	15.57 (<0.001)
Having children later in life	80 (14.6)	85 (15.5)	0.70 (0.484)	73 (13.1)	379 (67.8)	17.44 (<0.001)	0.75 (0.451)	17.60 (<0.001)
Early menstruation	69 (12.6)	77 (14.1)	1.16 (0.248)	61 (10.9)	365 (65.3)	17.10 (<0.001)	0.88 (0.380)	17.38 (<0.001)
Late menopause	87 (15.9)	95 (17.4)	1.21 (0.228)	79 (14.1)	376 (67.3)	17.23 (<0.001)	0.83 (0.409)	16.77 (<0.001)
Lack of physical activity	128 (23.4)	135 (24.7)	1.00 (0.336)	109 (19.5)	406 (72.6)	17.18 (<0.001)	1.60 (0.114)	15.94 (<0.001)
Total score ^#^ (mean ± SD)	2.16 ± 1.7	2.25 ± 1.6	1.87 ^c^ (0.061)	2.13 ± 1.7	6.72 ± 1.4	18.67 ^c^ (<0.001)	0.33 ^d^ (0.739)	27.27 ^d^ (<0.001)
**Warning symptom** ^~^								
Breast lump	188 (34.4)	192 (35.1)	0.94 (0.346)	220 (39.4)	427 (76.4)	14.12 (<0.001)	1.72 (0.086)	13.82 (<0.001)
Armpit lump	110 (20.1)	114 (20.8)	0.89 (0.371)	130 (23.3)	350 (62.6)	14.83 (<0.001)	1.27 (0.205)	14.07 (<0.001)
Nipple bleeding	158 (28.9)	160 (29.3)	0.50 (0.617)	186 (33.3)	424 (75.8)	15.43 (<0.001)	1.58 (0.115)	15.51 (<0.001)
Nipple pulling/retraction	104 (19.0)	110 (20.1)	1.50 (0.134)	131 (23.4)	415 (74.2)	16.74 (<0.001)	1.80 (0.072)	18.02 (<0.001)
Nipple position change	126 (23.0)	128 (23.4)	0.58 (0.564)	121 (21.6)	397 (71.0)	16.61 (<0.001)	0.55 (0.579)	15.85 (<0.001)
Nipple rash	139 (25.4)	142 (26.0)	0.58 (0.564)	140 (25.0)	436 (78.0)	17.21 (<0.001)	0.14 (0.888)	17.31 (<0.001)
Breast skin redness	127 (23.2)	130 (23.8)	0.83 (0.405)	135 (24.2)	411 (73.5)	16.61 (<0.001)	0.37 (0.715)	16.54 (<0.001)
Breast size change	134 (24.5)	137 (25.0)	1.13 (0.257)	136 (24.3)	428 (76.6)	17.09 (<0.001)	0.07 (0.948)	17.13 (<0.001)
Breast shape change	146 (26.7)	153 (28.0)	1.30 (0.194)	146 (26.1)	426 (76.2)	16.73 (<0.001)	0.22 (0.829)	16.05 (<0.001)
Breast pain	188 (34.4)	194 (35.5)	1.23 (0.221)	198 (35.4)	430 (76.9)	15.23 (<0.001)	0.37 (0.714)	13.90 (<0.001)
Breast puckering	84 (15.4)	90 (16.5)	1.50 (0.134)	87 (15.6)	413 (73.9)	18.06 (<0.001)	0.10 (0.924)	19.16 (<0.001)
Total score ^+^ (mean ± SD)	2.75 ± 2.55	2.83 ± 2.44	7.03 ^c^ (0.081)	2.92 ± 2.7	8.15 ± 1.88	20.01 ^c^ (<0.001)	0.83 ^d^ (0.408)	25.75 ^d^ (<0.001)

^^^ Counting only those who selected “agree”. ^#^ Total score was calculated by assigning one point for each risk factor correctly identified, with the total score ranging from 0 to 9. ^~^ Counting only those who selected “yes”. ^+^ Total score was calculated by assigning one point for each warning sign correctly identified, with the total score ranging from 0 to 11. ^a^ McNemar test. ^b^ Chi-squared test. ^c^ Wilcoxon signed-rank test. ^d^ Mann-Whitney U test. T0: pre-test; T1: post-test (4 weeks); Control: without education program; Intervention: with education program; HRT: hormone replacement therapy; SD: standard deviation.

**Table 3:** Comparison of barriers to seeking medical help and anticipated time to consult a doctor for breast cancer warning symptoms between groups at baseline (T0) and four weeks later (T1) (*N* = 1106).

	Control (n = 547)			Intervention (n = 559)			Control vs. Intervention	
	T0	T1	T0 vs. T1	T0	T1	T0 vs. T1	T0	T1
The Barrier to Seeking Medical Help ^^^	n (%)	n (%)	Test ^a^ (*p*-Value)	n (%)	n (%)	Test ^a^ (*p*-Value)	Test ^b^ (*p*-Value)	Test ^b^ (*p*-Value)
*Emotional barriers*								
Embarrassed	328 (60.0)	333 (60.9)	0.66 (0.508)	345 (61.7)	344 (61.5)	0.09 (0.926)	0.60 (0.550)	0.23 (0.822)
Scared	358 (65.4)	353 (64.5)	0.66 (0.508)	349 (62.4)	322 (57.6)	4.22 (<0.001)	4.22 (0.297)	2.36 (0.018)
Worried about what the doctor might find	339 (62.0)	342 (62.5)	0.45 (0.655)	343 (61.4)	316 (56.5)	3.08 (0.002)	0.21 (0.834)	2.03 (0.042)
Not confident talking about symptoms	309 (56.5)	306 (55.9)	0.60 (0.549)	295 (52.8)	288 (51.5)	0.86 (0.392)	1.24 (0.215)	1.47 (0.141)
*Practical barriers*								
Other things to worry about	242 (44.2)	240 (43.9)	0.32 (0.752)	234 (41.9)	209 (37.4)	3.20 (0.001)	0.80 (0.424)	2.20 (0.028)
Too busy	240 (43.9)	232 (42.4)	1.10 (0.258)	276 (49.4)	204 (36.5)	6.87 (<0.001)	1.83 (0.067)	2.01 (0.044)
Difficulty arranging transport	168 (30.7)	167 (30.5)	0.13 (0.900)	162 (29.0)	140 (25.0)	3.24 (0.001)	0.63 (0.529)	2.17 (0.030)
*Service barriers*								
Difficulty making an appointment	174 (31.8)	179 (32.7)	0.80 (0.423)	177 (31.7)	174 (31.1)	0.66 (0.513)	0.05 (0.958)	0.57 (0.568)
Worried about wasting the doctor’s time	92 (16.8)	93 (17.0)	0.14 (0.889)	81 (14.5)	65 (11.6)	3.41 (0.001)	1.07 (0.287)	2.55 (0.011)
Difficulty talking to the doctor	305 (55.8)	301 (55.0)	0.89 (0.371)	306 (54.7)	272 (48.7)	4.25 (<0.001)	0.34 (0.734)	2.12 (0.034)
Total score ^#^ (mean ± SD)	4.67 ± 2.6	4.65 ± 2.4	0.30 ^c^ (0.765)	4.59 ± 2.5	4.17 ± 2.2	7.91 ^c^ (<0.001)	0.57 ^d^ (0.569)	3.44 ^d^ (0.001)
**Promptly consulting a doctor per warning symptom** ^~^								
Breast lump	250 (45.7)	243 (44.4)	0.60 (0.553)	266 (47.6)	487 (87.1)	13.62 (<0.001)	1.46 (0.144)	15.84 (<0.001)
Armpit lump	225 (41.1)	230 (42.0)	1.25 (0.211)	239 (42.8)	453 (81.0)	13.17 (<0.001)	1.70 (0.089)	14.05 (<0.001)
Nipple bleeding	309 (56.5)	312 (57.0)	0.99 (0.320)	331 (59.2)	514 (91.9)	12.34 (<0.001)	1.60 (0.110)	13.80 (<0.001)
Nipple pulling/retraction	189 (34.6)	195 (35.6)	1.46 (0.146)	190 (34.0)	426 (76.2)	14.20 (<0.001)	0.51 (0.607)	14.41 (<0.001)
Nipple position change	206 (37.7)	215 (39.3)	1.84 (0.066)	198 (35.4)	434 (77.6)	14.20 (<0.001)	0.84 (0.339)	14.16 (<0.001)
Nipple rash	242 (44.2)	234 (42.8)	0.92 (0.357)	253 (45.3)	451 (80.7)	12.71 (<0.001)	1.47 (0.142)	13.74 (<0.001)
Breast skin redness	215 (39.3)	212 (38.8)	0.19 (0.851)	228 (40.8)	384 (68.7)	11.22 (<0.001)	1.51 (0.131)	10.57 (<0.001)
Breast size change	150 (27.4)	152 (27.8)	1.43 (0.152)	150 (26.8)	374 (66.9)	13.65 (<0.001)	0.76 (0.447)	13.61 (<0.001)
Breast shape change	177 (32.4)	176 (32.2)	0.42 (0.672)	179 (32.0)	373 (66.7)	12.70 (<0.001)	0.77 (0.444)	12.36 (<0.001)
Breast pain	246 (45.0)	247 (45.2)	0.01 (0.919)	264 (47.2)	412 (73.7)	10.89 (<0.001)	1.88 (0.061)	14.44 (<0.001)
Breast puckering	168 (30.7)	163 (29.8)	1.60 (0.112)	180 (32.2)	375 (67.1)	12.74 (<0.001)	1.44 (0.149)	12.87 (<0.001)
Total score ^+^ (mean ± SD)	33.27 ± 16.8	33.49 ± 15.8	1.87 ^c^ (0.061)	35.03 ± 15.8	48.29 ± 5.9	18.67 ^c^ (<0.001)	1.46 ^d^ (0.145)	15.78 ^d^ (<0.001)

^^^ Counting only those who selected “yes”. ^#^ Total score was calculated by assigning one point for each barrier identified, with the total score ranging from 0 to 10. ^~^ Counting only those who selected “within the first two weeks” for each symptom. ^+^ Total score was calculated by assigning five points for each symptom marked “within the first two weeks”, with the total score ranging from 11 to 55. ^a^ McNemar test. ^b^ Chi-squared test. ^c^ Wilcoxon signed-rank test. ^d^ Mann-Whitney U test. T0: pre-test; T1: post-test (4 weeks); Control: without education program; Intervention: with education program; SD: standard deviation.

## Data Availability

The datasets are unavailable online due to privacy or ethical restrictions. Data are available from the corresponding author upon reasonable request.
